# Nervonic acid supplementation mitigates disease severity biomarkers in adrenoleukodystrophy

**DOI:** 10.1016/j.neurot.2026.e00890

**Published:** 2026-04-07

**Authors:** Chenxu Li, Kai Braaten, Ann B. Moser, Wedad Fallatah, Maggie Lorentson, Suzette Huguenin, Anna Torrey, Marcia R. Terluk, Willa Durose, Erin Nolan, Christopher Staley, Subbaya Subramanian, Troy C. Lund, Reena V. Kartha

**Affiliations:** a Center for Orphan Drug Research, University of Minnesota, Minneapolis, MN, USA; b Department of Experimental and Clinical Pharmacology, College of Pharmacy, University of Minnesota, Minneapolis, MN, USA; c Division of Pediatric Blood and Marrow Transplant, University of Minnesota, Minneapolis, MN, USA; d Genetics Laboratories, Kennedy Krieger Institute, Baltimore, MD, USA; e Department of Genetic Medicine, Johns Hopkins University, Baltimore, MD, USA; f Department of Surgery, Medical School, University of Minnesota, Minneapolis, MN, USA

**Keywords:** Adrenoleukodystrophy, Nervonic acid, Very long-chain fatty acids, C26:0-lysophosphatidylcholine, Microbiome, Dietary therapy

## Abstract

X-linked adrenoleukodystrophy (ALD) is a severe neurometabolic disorder caused by mutations in the *ABCD1* gene, leading to impaired peroxisomal β-oxidation of very long-chain fatty acids (VLCFAs). The accumulation of saturated VLCFAs, predominantly C26:0, in plasma and across all tissues, contributes to adrenal dysfunction and progressive neurodegeneration. No approved therapy addresses the diverse spectrum of ALD manifestations, underscoring the urgent need for safe, accessible, and preventive treatments. Nervonic acid (NA), a monounsaturated fatty acid, is potentially beneficial for ALD through its neuroprotective effects. Here, we report the safety and therapeutic efficacy of NA in a 4-week dietary intervention study using a mouse model of ALD. NA treatment significantly decreased plasma C26:0-lysophosphatidylcholine, a diagnostic and disease-severity biomarker of ALD, by about 60% as early as one week after intervention. After 4-week treatment, NA markedly reduced free C26:0 and total saturated VLCFA levels in plasma and tissues. Moreover, we observed approximately 56% reduction in brain C26:0-lysophosphatidylcholine levels in NA-fed mice, an effect not reported with other drug intervention. Through comparative microbiome analysis, we show for the first time distinct baseline differences between ALD and wild-type mice, with dietary fatty acid supplementation preventing further dysbiosis. No adverse effects on body weight or food intake were observed throughout the study. Overall, this is the first report demonstrating that an oral dietary fatty acid can ameliorate the hallmark biochemical abnormalities of ALD in plasma and brain, highlighting its potential as a safe and effective therapy, particularly for presymptomatic individuals carrying this genetic defect.

## Introduction

X-linked adrenoleukodystrophy (ALD) is a rare metabolic disorder caused by mutations in the *ABCD1* gene, which encodes an ATP-binding cassette transporter [1]. This protein, located on the peroxisomal membrane, is responsible for transporting very long-chain fatty acids (VLCFAs) into peroxisomes for β-oxidation [2]. Mutations in this gene result in a loss of protein function, leading to the accumulation of saturated VLCFAs, predominantly hexacosanoic acid (C26:0), in plasma, brain white matter, spinal cord, adrenal glands, and testes [3]. This biochemical defect triggers a cascade of downstream pathological events, including oxidative stress, mitochondrial impairment, calcium dysregulation, bioenergetic failures, neuroinflammation, demyelination, and axonal degeneration, all of which contribute to disease progression [2,[4], [5], [6]].

ALD manifests as a broad spectrum, ranging from isolated adrenal insufficiency to adrenomyeloneuropathy (AMN) and the most severe and progressive form, cerebral ALD (cALD), which leads to rapid neurological decline and even death within a few years [7]. Although males are more severely affected, more than 80% of females develop spinal cord disease in adulthood [8]. Clear genotype-phenotype correlations are not established, and clinical features frequently overlap, with adrenal insufficiency often accompanying other phenotypes, and ∼20% of AMN cases progressing to cerebral demyelination [7,9]. The heterogeneity and unpredictability of ALD underscore the need for early diagnosis and timely intervention, particularly for individuals at risk of developing severe neurological manifestations. Current treatment options, including allogeneic hematopoietic stem cell transplantation and lentiviral gene therapy, are limited to early-stage cALD and carry substantial risks, such as graft failure, graft-versus-host disease, transplant-related mortality, and insertional oncogenesis [[10], [11], [12]]. No FDA-approved therapy addresses the core biochemical defect of ALD or prevents symptom onset, underscoring the urgent need for safe and broadly effective treatments across the phenotypic spectrum.

We have previously shown that nervonic acid (NA, C24:1), a naturally occurring monounsaturated fatty acid, can biochemically reverse the accumulation of C26:0 and total saturated VLCFAs, enhance mitochondrial function, and reduce cellular oxidative stress in ALD patient-derived fibroblasts [13,14]. Literature evidence also indicates that NA exerts protective effects against neuroinflammation, cognitive decline, motor dysfunction, neurological deficits, and demyelination by regulating multiple transcriptional and metabolic pathways [[15], [16], [17], [18]]. Here, we hypothesize that NA can reroute metabolism by promoting the formation of monounsaturated fatty acids rather than the toxic saturated forms. To validate our in vitro observations and to test our hypothesis, we designed a rigorously controlled dietary intervention study with NA in a well-established ALD mouse model [19]. The primary objective was to evaluate the biochemical effects of NA on diagnostic and disease severity biomarkers of ALD. In addition, gastrointestinal disturbances are common in individuals with leukodystrophies, and increasing evidence links gut microbiota alterations with neurodegenerative disorders [20]. Accordingly, a secondary objective was to assess the impact of dietary fatty acid supplementation on gut microbiome composition.

## Materials and Methods

### Materials

Nervonic acid (≥99%, capillary GC) and erucic acid (≥99%, capillary GC-MS) were purchased from Sigma-Aldrich (Burlington, MA, USA). Teklad Rodent Chow #2018 supplemented with NA or erucic acid (EA, as a comparator) was custom-formulated and purchased from Research Diets, Inc. (New Brunswick, NJ, USA). SAFE-T-FILL™ Micro Capillary Blood Collection Tubes were purchased from Fisher Scientific (Waltham, MA, USA). Glass screw-capped tubes, Eppendorf Safe-Lock tubes, stainless steel beads, Pasteur glass pipettes with rubber bulbs, Wheaton (Socorex) micropipettes with disposable glass capillary tubes were purchased from Fisher Scientific (Hanover Park, IL, USA). Chloroform was obtained from Honeywell-Burdick & Jackson (Muskegon, MI, USA). Methanol, pyridine, hexane, ethyl acetate, 95% ethanol (all Optima-grade solvents), potassium hydroxide, and butylated hydroxytoluene (BHT) were purchased from Fisher Chemicals (Fairlawn, NJ, USA). Milli-Q water was produced in the laboratory with a purity of 18.2 megohms. Standard fatty acids were purchased from Sigma-Aldrich (Burlington, MA, USA) and verified for purity by gas chromatography (GC) prior to use.

### Mouse model and dietary treatment

The *Abcd1−/−* mouse model, developed by the Kennedy Krieger Institute, exhibits biochemical abnormalities comparable to those observed in patients with ALD. This model demonstrates impaired peroxisomal β-oxidation of VLCFAs, resulting in markedly elevated levels of saturated VLCFAs in various tissues, including the brain and adrenal glands [19]. Starting at 2 months of age, *Abcd1−/−* male mice (n = 4 per group; N = 16) were randomly assigned to experimental groups and fed Teklad Rodent Chow #2018 supplemented with either 0.6% NA (1 g/kg), 3% NA (5 g/kg), or 3% EA (5 g/kg) for 28 days, with doses expressed in g/kg body weight/day. Mice receiving unsupplemented Teklad Rodent Chow #2018 served as the control group. In addition, age-matched wild-type C57BL/6J mice (n = 4) fed with standard Teklad Rodent Chow #2018 were included as wild-type controls. Sample size was determined based on literature reporting similar endpoints [21] and on institutional animal-use considerations, with the aim of minimizing animal numbers while preserving the ability to detect biologically meaningful effects. All mice were housed on a 14:10 light-dark cycle and maintained at a temperature not exceeding 24 °C, with *ad libitum* access to food and water at the Research Animal Resources facility, University of Minnesota. All procedures were approved by the Institutional Animal Care and Use Committee of the University of Minnesota (IACUC protocol #2107–39290A) prior to study commencement. This study complies with the ARRIVE guidelines and was conducted in accordance with the U.S. National Research Council's Guide for the Care and Use of Laboratory Animals. The study design is illustrated in the schematic diagram shown in Fig. 1A.

**Fig. 1:**
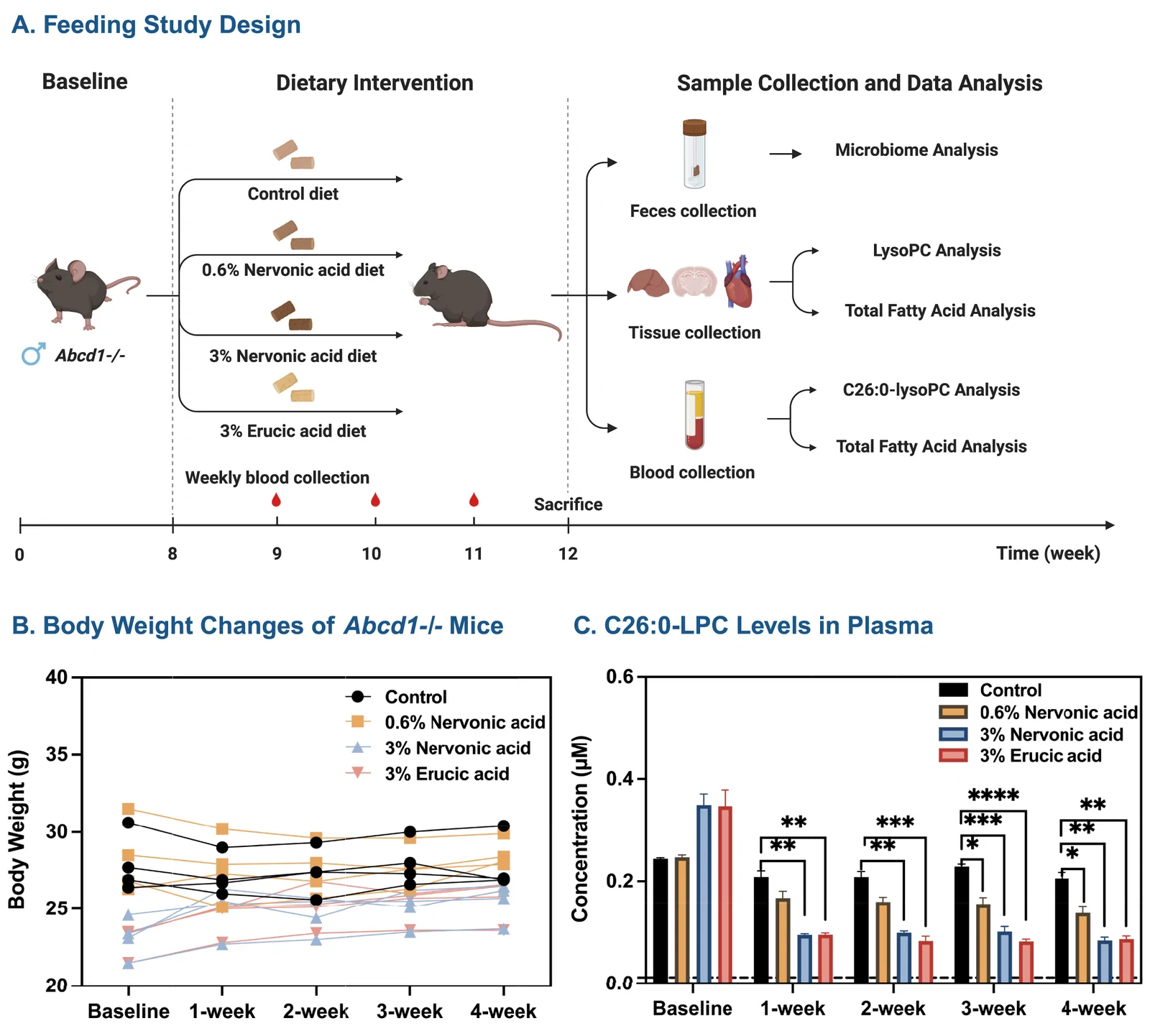
Overview of study design, longitudinal body weight changes, and changes in plasma C26:0-LPC levels with dietary intervention. **(A)** Schematic representation of the feeding study design. Eight-week-old male *Abcd1−/−* mice were fed customized diets for four weeks, including a control chow and chows supplemented with either 0.6% or 3% nervonic acid or 3% erucic acid. **(B)** Weekly body weight changes were monitored for each mouse from baseline at 8 weeks of age through the end of the study at 12 weeks of age. **(C)** Concentrations of C26:0-LPC in plasma were measured on a weekly basis. Treatment effects at each time point were analyzed using two-way ANOVA followed by Tukey's test. The dashed line represents the average plasma C26:0-LPC level observed in wild-type mice. Data are represented as mean ± SEM (n = 4 per group). ∗, p < 0.05; ∗∗, p < 0.01; ∗∗∗, p < 0.001; ∗∗∗∗, p < 0.0001.

### Sample collections

Blood samples were collected at baseline (predose) and at 1, 2, 3, and 4 weeks following the initiation of dietary intervention. At baseline and at the 4-week endpoint, sufficient plasma volumes were collected to enable both C26:0-lysophosphatidylcholine quantification and total fatty acid analysis. For the interim time points (1, 2, and 3-week sampling), only small blood volumes (∼60 μL) were collected via the cheek vein, as the high sensitivity of the C26:0-LPC assay enabled reliable quantification from minimal sample volumes, thereby minimizing animal stress and blood loss. Whole blood was collected into EDTA-coated microcapillary collection tubes and immediately centrifuged at 2000×*g* for 15 min to separate plasma, which was then stored at −80 °C until analysis. At the end of the study, mice were euthanized and tissue samples including the whole brain, liver, kidney, heart, and adrenal gland were collected, immediately flash-frozen in liquid nitrogen, and stored at −80 °C until analysis. Fecal samples were collected at baseline (predose) and at 1, 2, 3, and 4 weeks after treatment initiation. During collection, mice were transferred to individual autoclaved cages with water but without food or bedding and isolated for 1 h to collect individual fecal samples. Fecal pellets were collected using a sterilized tweezer and placed into pre-labeled Eppendorf tubes. On average, 3 to 5 pellets were obtained per mouse. Following collection, mice were returned to their original cages, and samples were stored at −80 °C until processing for DNA isolation.

### Total lipid extraction from plasma and tissues

Total lipids were extracted from frozen plasma and tissue samples as previously described [22]. Briefly, frozen tissues were weighed and homogenized in 1 mL of methanol using the Qiagen TissueLyser, followed by extraction with 2 mL of chloroform and 2 mL of 2:1 chloroform: methanol solutions for at least 1 h. After phase separation by centrifugation (800×g, 10 min), the organic supernatant was carefully collected into a clean pre-weighed tube. The remaining pellet was re-extracted with 1 mL of 2:1 chloroform: methanol, vortexed, centrifuged, and the second supernatant was combined with the first. The combined organic layers were dried under nitrogen, and the lipid residue was desiccated under vacuum for 30 min. Tubes were then reweighed to determine total lipid content and stored capped at −30 °C until further analysis.

### Quantification of total lipid fatty acids using negative ion GC/MS

An aliquot of about 2,000–3,000 μg of total lipid extract or 50–100 μL of plasma was transferred into screw-cap glass tubes containing 200 μL of BHT (50 mg/L in isopropanol: hexane, 2:3). 50 μL of a combined internal standard solution containing C27:0 and C19:0 fatty acids in chloroform (0.02 mg/mL) was added, along with 100 μL of the deuterium-labeled fatty acid mix, to each sample. Samples were dried under nitrogen and stored at −25 °C until analysis. Fatty acids were derivatized to pentafluorobenzyl esters and quantified by negative ion GC/MS using an Agilent 7890A GC system coupled to a 5975C mass selective detector, with helium as the carrier gas and ammonia as the reagent gas. Separation was performed on a Supelco SP-2530 column (50 m × 0.25 mm i.d. × 0.25 μm film thickness). This method is adapted from a previously published protocol [23].

### Quantification of lysophosphatidylcholine (LPC) using LC-MS/MS

LPC species were quantified in mouse plasma and tissues using LC-MS/MS, following a modified protocol based on previously published literature [[24], [25], [26]]. Aliquots of 10 μL plasma were extracted in 150 μL of methanol containing deuterated LPC internal standards (C20:0-C26:0, D4-labeled), followed by vortexing, incubation, and centrifugation. Supernatants were filtered and transferred to autosampler vials for analysis. An 8-point calibration curve, along with normal controls, abnormal controls, and quality control samples (including CDC reference samples), were processed in parallel. Chromatographic separation was performed on a reverse-phase Kinetex C8 column (50 × 2.1 mm, 2.6 μm) using a gradient of 0.1% formic acid in water (A) and methanol (B) at 0.35 mL/min. Data were processed using Analyst software, with quantification based on calibration curves (R^2^ > 0.980) and reported as pmol/mL for plasma samples.

### DNA extraction and 16S rRNA sequencing

DNA was extracted from fecal samples using the DNeasy PowerSoil Kit, purchased from QIAGEN (Germantown, MD, USA), with the QIACube automated platform following the manufacturer's protocol. The V4 region of the 16S rRNA gene was amplified and sequenced using the 515F/806R primer set by the University of Minnesota Genomics Center (UMGC). Paired-end sequencing was done at a read length of 301 bp on the Illumina MiSeq platform using a dual-index approach by UMGC. Raw sequence data were deposited in the NCBI Sequence Read Archive under BioProject accession number SRP565277.

### 16S rRNA amplicon processing and annotation

Sequence data were processed and analyzed using Mothur [27] (v1.41.1) and our previously published pipeline [28], modified for the V4 region. Briefly, sequences were trimmed to 170 nt and paired-end joined using fastq-join software. Sequences were trimmed for quality [homopolymers ≤6; no ambiguous bases (N); sliding window size = 50; quality score ≥35; ≤2 mismatches from primer sequences] and aligned against the SILVA database [29] (v138.1). Aligned sequences were filtered for the primer region, subjected to a 2% pre-cluster to remove likely errors, and chimeras were identified and removed using UCHIME [30] (v4.2.40). Sequences were clustered using *OptiClust* [31], with amplicon sequence variants binned at 99% similarity. Taxonomic annotations were made against the Ribosomal Database Project [32] (v18). Samples were normalized to 5800 reads per sample for statistical analyses.

### Statistical analyses

For free fatty acid and complex lipid analyses, data were plotted and statistical analyses were performed using GraphPad Prism, v10.1.2 (GraphPad Software, LLC, San Diego, CA, USA). Two-way analysis of variance (ANOVA) was conducted to assess the effects of treatment and time on fatty acid and complex lipid levels across groups. Post hoc analysis using Tukey's Honestly Significant Difference (HSD) test was performed to adjust for multiple comparisons. A p-value of less than 0.05 was considered statistically significant.

For microbiome analysis, within-sample (α) and between-sample (β) diversity were calculated in Mothur. α-diversity was determined from the Shannon and Chao1 indices, while β-diversity was evaluated using Bray-Curtis dissimilarity matrices. Differences in α-diversity were evaluated by ANOVA with Tukey's post hoc test using XLSTAT (v2020.2.3; Addinsoft, Belmont, MA, USA), with a covariance matrix correction to meet the assumptions of normality. Differences in abundances of taxa were determined using linear discriminant analysis (LDA) of effect size [33] (LefSe). Differences in β-diversity were determined using analysis of similarity [34] (ANOSIM). Longitudinal differences in abundances of genera (or the most resolved taxonomic level available) were determined using the permuspliner function in SplinectomeR implemented in R [35] (v4.4.0). All statistics were evaluated at an experimental α = 0.05, with Bonferroni correction for multiple comparisons.

## Results

### Monitoring of body weight and daily food consumption

Individual mice were weighed weekly to monitor body weight changes, as shown in Fig. 1B. Daily administration of NA or EA had no significant effect on body weight, with measured values comparable to the reference body weights reported by the Jackson Laboratory for C57BL/6J male mice [36]. In addition, daily food intake was recorded for each mouse. There were no significant differences in average daily food consumption across the groups. The average daily food consumption was 3.23 g for the control group, 3.18 g for the 0.6% NA group, 3.07 g for the 3% NA group, and 3.30 g for the 3% EA group. Notably, no significant abnormalities were observed in tissues collected from the NA-treated groups at the end of the study. In contrast, one mouse in the EA group exhibited an enlarged heart.

### Nervonic acid significantly reduced plasma C26:0-lysophosphatidylcholine levels in *Abcd1−/−* mice

Plasma C26:0-LPC levels were measured and quantified every week throughout the treatment period and are presented in Fig. 1C. Treatment with either NA or EA at 3% of the daily diet resulted in a significant reduction in plasma C26:0-LPC levels as early as 1 week after treatment initiation. Notably, this reduction was sustained throughout the 4-week duration of the study. Moreover, a dose-dependent response to NA was observed. Unlike the 3% diet, mice fed on the 0.6% NA diet exhibited a statistically significant reduction in plasma C26:0-LPC levels only at 3 weeks after treatment initiation, which was then maintained until the end of the study. By the end of the treatment period, mice in the 3% NA group showed approximately a 60% reduction in plasma C26:0-LPC levels compared to the control group. While this reduction did not fully restore C26:0-LPC levels to the normal values observed in age-matched wild-type mice (∼0.01 μM, indicated by the dashed line), these observations indicate NA to be an effective modulator of VLCFA metabolism by significantly lowering circulating C26:0-LPC, a key diagnostic biomarker of ALD.

### Nervonic acid significantly decreased total plasma C26:0 and total saturated VLCFA levels in *Abcd1−/−* mice

The total plasma lipid profile was measured at baseline (before treatment) and after 4 weeks of dietary intervention. At the study endpoint, mice treated with 3% NA or 3% EA exhibited significantly reduced plasma levels of free C26:0 and total saturated VLCFAs compared to the control group (Fig. 2A, G). However, mice fed with 0.6% NA diet demonstrated a significant reduction only in total saturated VLCFAs after 4 weeks of treatment. Importantly, both doses of NA reduced total saturated VLCFA levels to below those observed in wild-type mice, similar to the reductions seen in mice fed the EA diet, indicating a robust therapeutic effect. Additionally, analysis of the ratios of C26:0/C22:0 and C24:0/C22:0 indicated a significant reduction in the C24:0/C22:0 ratio only in the EA treatment group, while both NA and EA treatments resulted in an elevated C26:0/C22:0 ratio (Table 1). These ratio changes, however, are primarily attributed to differential reductions among the individual fatty acids, specifically a greater decrease in C22:0 relative to C26:0 and C24:0. Notably, all three fatty acids were significantly reduced following the interventions (Fig. 2A–C). Moreover, plasma levels of monounsaturated VLCFAs, including C24:1 (NA), C22:1 (EA), and C26:1 were quantified (Fig. 2D–F). Treatment with NA or EA resulted in significant increases in both C24:1 and C26:1, supporting the hypothesis that these interventions promote metabolic rerouting from saturated to monounsaturated VLCFA synthesis. However, only EA-fed mice showed a significant increase in C22:1, consistent with impaired β-oxidation observed in ALD. Collectively, these findings demonstrate the efficacy of NA in reducing plasma saturated VLCFAs, a hallmark metabolic disturbance in ALD, and provide further mechanistic insight into its therapeutic potential.

**Fig. 2:**
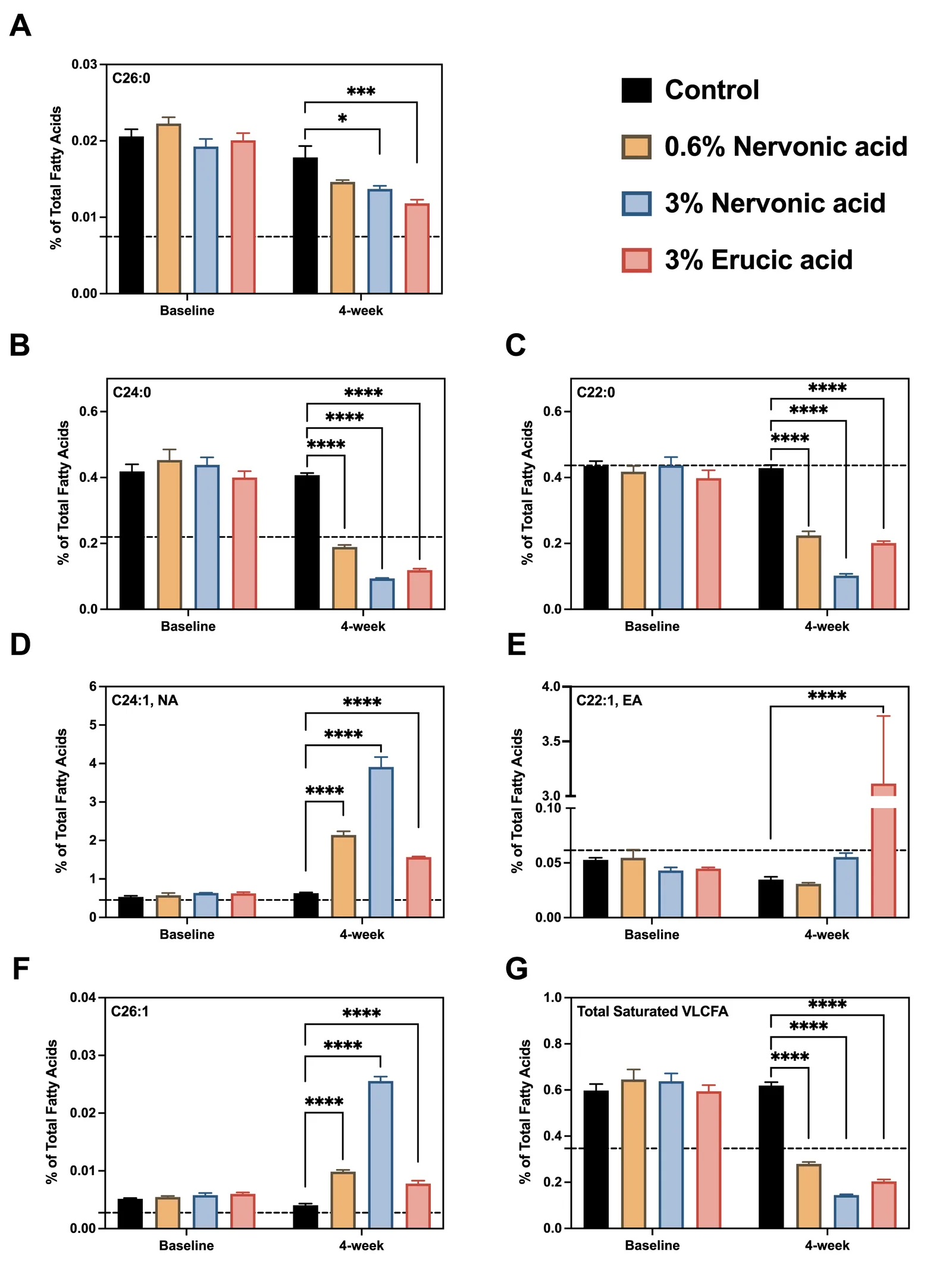
Plasma fatty acid profiles in *Abcd1−/−* mice at baseline and after 4 weeks of dietary interventions. Fatty acid levels were quantified as a percentage of total fatty acids. Plasma levels of: **(A)** C26:0, **(B)** C24:0, **(C)** C22:0, **(D)** C24:1 (NA), **(E)** C22:1 (EA), **(F)** C26:1, and **(G)** total saturated VLCFAs are shown. Treatment effects were analyzed using two-way ANOVA followed by Tukey's post hoc test. The dashed lines represent the average fatty acid levels observed in wild-type mice. Data are represented as mean ± SEM (n = 4 per group). ∗, p < 0.05; ∗∗, p < 0.01; ∗∗∗, p < 0.001; ∗∗∗∗, p < 0.0001.

**Table 1:** Plasma C26:0/C22:0 and C24:0/C22:0 ratios in *Abcd1−/−* mice following 4-week treatment.

	Control		0.6 % Nervonic acid		3% Nervonic acid		3% Erucic acid		Wild-type
	Baseline	4-week	Baseline	4-week	Baseline	4-week	Baseline	4-week	Baseline
C26:0/C22:0	0.047 ± 0.002	0.042 ± 0.003	0.054 ± 0.003	0.066 ± 0.004 ∗∗∗	0.044 ± 0.003	0.135 ± 0.006 ∗∗∗∗	0.051 ± 0.005	0.059 ± 0.001 ∗	0.0172 ± 0.005
C24:0/C22:0	0.960 ± 0.028	0.951 ± 0.020	1.083 ± 0.043	0.849 ± 0.042	1.005 ± 0.028	0.920 ± 0.039	1.007 ± 0.033	0.592 ± 0.012 ∗∗∗∗	0.505 ± 0.043

Data are represented as mean ± SEM (n = 4 per group). The asterisk (∗) indicates a statistically significant difference between the treatment groups and the control group at the end of the 4-week study. ∗, p < 0.05; ∗∗, p < 0.01; ∗∗∗, p < 0.001; ∗∗∗∗, p < 0.0001.

### Nervonic acid significantly diminished total lipid C26:0 and total saturated VLCFA levels in tissues in *Abcd1−/−* mice

Total fatty acid profiles in tissues, including the liver, kidney, heart, brain, and adrenal gland, were quantified at the end of 4 weeks of dietary intervention. We observed a dose-dependent effect of NA on free C26:0 and total saturated VLCFAs in the kidney and brain. Specifically, the 3% NA diet significantly reduced total saturated VLCFAs, including C26:0, similar to the effect observed with 3% EA. Furthermore, total saturated VLCFA levels in the liver were also significantly decreased by NA treatment, similar to the reduction observed with EA. However, no significant changes in C26:0 or total saturated VLCFAs were observed in the adrenal gland (Fig. 3A, E).

**Fig. 3:**
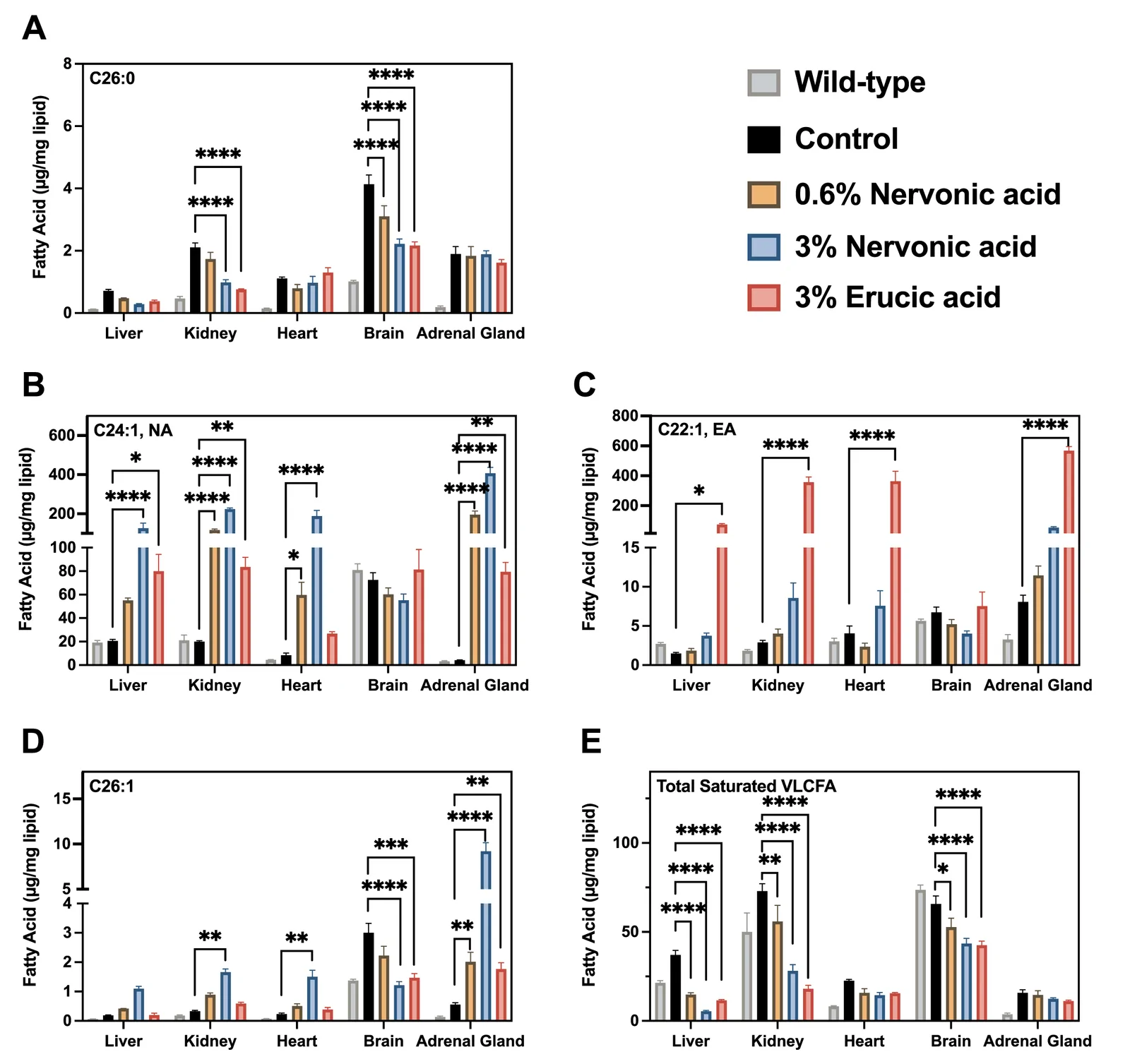
Fatty acid profiles in the liver, kidney, heart, brain, and adrenal gland of *Abcd1−/−* mice after 4 weeks of dietary interventions. Concentrations of **(A)** C26:0, **(B)** C24:1 (NA), **(C)** C22:1 (EA), **(D)** C26:1, and **(E)** total saturated VLCFAs in different tissues are shown in μg/mg lipid. Treatment effects were analyzed using two-way ANOVA followed by Tukey's post hoc test. Data are represented as mean ± SEM (n = 4 per group). ∗, p < 0.05; ∗∗, p < 0.01; ∗∗∗, p < 0.001; ∗∗∗∗, p < 0.0001.

Consistent with the proposed metabolic rerouting hypothesis, C26:1 levels were significantly elevated in the kidney, heart, and adrenal gland following treatment (Fig. 3D). Interestingly, unlike in other tissues, brain C26:1 levels were significantly reduced to wild-type levels following treatment with either 3% NA or EA. As expected, we observed a dose-dependent increase in C24:1 levels following NA diets in the liver, kidney, heart, and adrenal glands. Likewise, EA treatment also resulted in marked elevation of C24:1 levels across these tissues, indicating normal fatty acid chain elongation (Fig. 3B). In contrast, elevated C22:1 levels were observed in the liver, kidney, heart, and adrenal gland only in the EA-treated group, consistent with the findings in the plasma (Fig. 3C). Notably, the brain levels of both C24:1 and C22:1 exhibited a decreasing trend following NA treatment, although the changes were not statistically significant.

Overall, our findings demonstrate that NA effectively attenuates free saturated VLCFA accumulation across multiple tissues, including the brain, thereby providing biochemical evidence supporting its therapeutic efficacy in addressing VLCFA-associated pathologies.

### Nervonic acid significantly attenuated the accumulation of C26:0-lysophosphatidylcholine in the brain of *Abcd1−/−* mice

Given the significant reduction in plasma C26:0-LPC levels following NA treatment, we investigated whether a similar trend could be observed in tissues. At baseline, among the different tissues, brains from *Abcd1−/−* mice showed an average 4.5-fold increase in C26:0-LPC compared to wild-type mice. Following 4 weeks of fatty acid diet, consistent with our findings in plasma, brain C26:0-LPC levels were significantly decreased by both doses of NA, similar to EA (Fig. 4A). Among all treatment groups and tissues tested, 3% NA exhibited the most pronounced effect in the brain, reducing C26:0-LPC levels by approximately 56% compared to controls. Treatment with either NA or EA led to significant and dose-dependent increases in C24:1-LPC levels in the liver, kidney, and heart (Fig. 4B). In parallel, elevated C26:1-LPC levels were observed in the liver and kidney, corresponding to the changes in C24:1-LPC (Fig. 4C). In contrast, brain C24:1-LPC levels showed a decreasing trend, while C26:1-LPC levels were significantly reduced following treatment with 3% NA or EA, similar to the changes observed in free fatty acids. These findings further demonstrate that NA can modulate both free fatty acid concentrations and their corresponding complex lipid species across various tissues, with particularly pronounced effects observed in the brain.

**Fig. 4:**
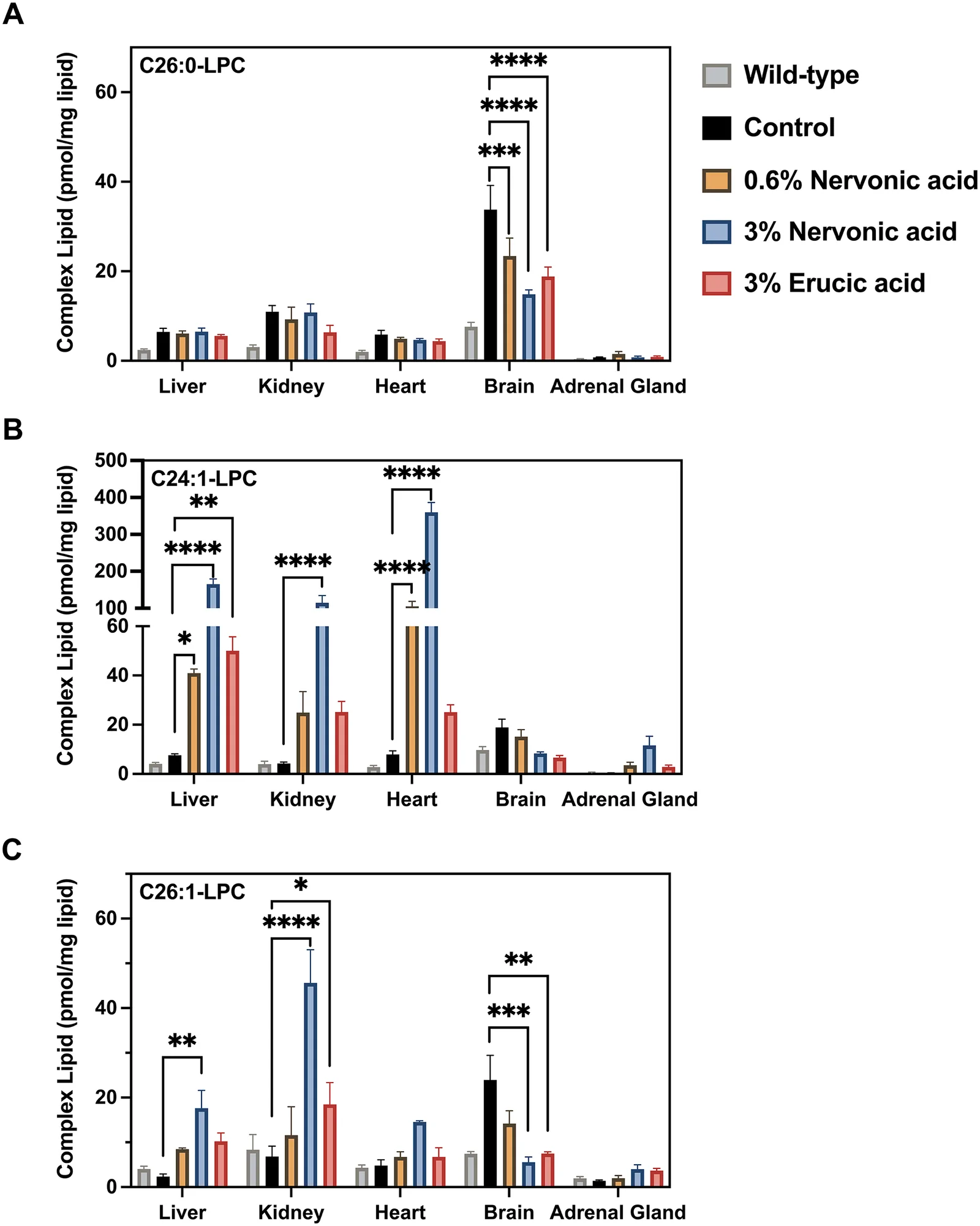
Lysophosphatidylcholine (LPC) species in the liver, kidney, heart, brain, and adrenal gland of *Abcd1−/−* mice after 4 weeks of dietary intervention. Concentrations of **(A)** C26:0-LPC, **(B)** C24:1-LPC, and **(C)** C26:1-LPC in different tissues are shown. Treatment effects were analyzed using two-way ANOVA followed by Tukey's post hoc test. Data are represented as mean ± SEM (n = 4 per group). ∗, p < 0.05; ∗∗, p < 0.01; ∗∗∗, p < 0.001; ∗∗∗∗, p < 0.0001.

### *Abcd1−/−* mice show altered fecal microbial profiles compared to wild-type mice

Next, we examined whether dietary interventions could alter the gut microbiota. First, we compared the baseline microbial profiles of *Abcd1−/−* and wild-type mice. Among all samples, a mean of 99.0 ± 0.5% estimated Good's coverage was achieved at a rarefaction depth of 5800 reads per sample. A median of 193 ASVs (interquartile range 177–219) were observed. At baseline, fecal microbial community structure (β-diversity) of *Abcd1−/−* mice was significantly different from that of wild-type animals (ANOSIM R = 1.00, p = 0.034; Fig. 5A), where *Abcd1−/−* mice had greater abundances of members of the genus *Faecalibaculum* while wild-type mice harbored greater abundances of *Lactobacillus* (Fig. 5A, Supplementary Table 1). No significant differences in community structure were observed longitudinally among *Abcd1−/−* mice (ANOSIM R = 0.059, p = 0.272; Fig. 5B). Similarly, no significant differences were observed in either the Shannon (Fig. 5C) or Chao1 (data not shown) indices over time (F = 1.70 and 1.10; p = 0.202 and 0.391).

**Fig. 5:**
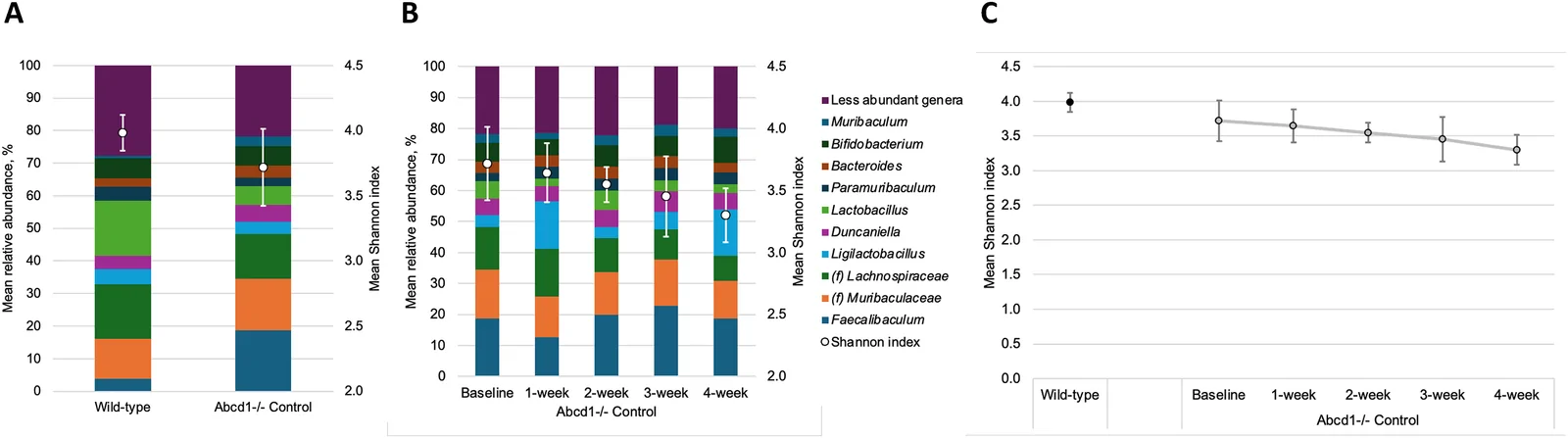
Comparison of wild-type and *Abcd1−/−* mice. **(A)** Baseline distribution of predominant genera (n = 4 per group), **(B)** Longitudinal distribution of genera in *Abcd1−/−* mice (n = 4 per time point). **(C)** Shannon indices for wild-type and *Abcd1−/−* mice. Wild-type mice were only sampled at baseline. Less abundant genera were consolidated for clarity of presentation. Error bars reflect standard deviation. (f) indicates taxon which could not be further classified beyond the family.

### Dietary fatty acid intake prevented further dysbiosis in *Abcd1−/−* mice

We then characterized gut microbiota in *Abcd1−/−* mice following dietary interventions. At baseline and following treatment with either NA or EA, the profiles showed similar Shannon indices, which tended to decline over the duration of the study. While Shannon indices did differ significantly among all treatments and time points (ANOVA F = 2.01, p = 0.020; Fig. 6A), pairwise comparisons were not significant by Tukey's post hoc test (p ≥ 0.355). Differences in Chao1 indices among *Abcd1−/−* mice were not significantly different (F = 1.136, p = 0.340; data not shown). Similarly, while differences in community structure (β-diversity) among treatments at baseline were significant for the experimental comparison (ANOSIM R = 0.784, p < 0.001; Fig. 6B, Supplementary Table 1), pairwise comparisons did not reach statistical significance at Bonferroni-adjusted α = 0.005; p ≥ 0.029. At the endpoint, pairwise differences in community structure between treatment groups also failed to reach statistical significance at Bonferroni-adjusted α = 0.008; p ≥ 0.027. The greatest differences were observed between the control and those treated with 3% EA (R = 0.74, p = 0.027; Supplementary Table 2).

**Fig. 6:**
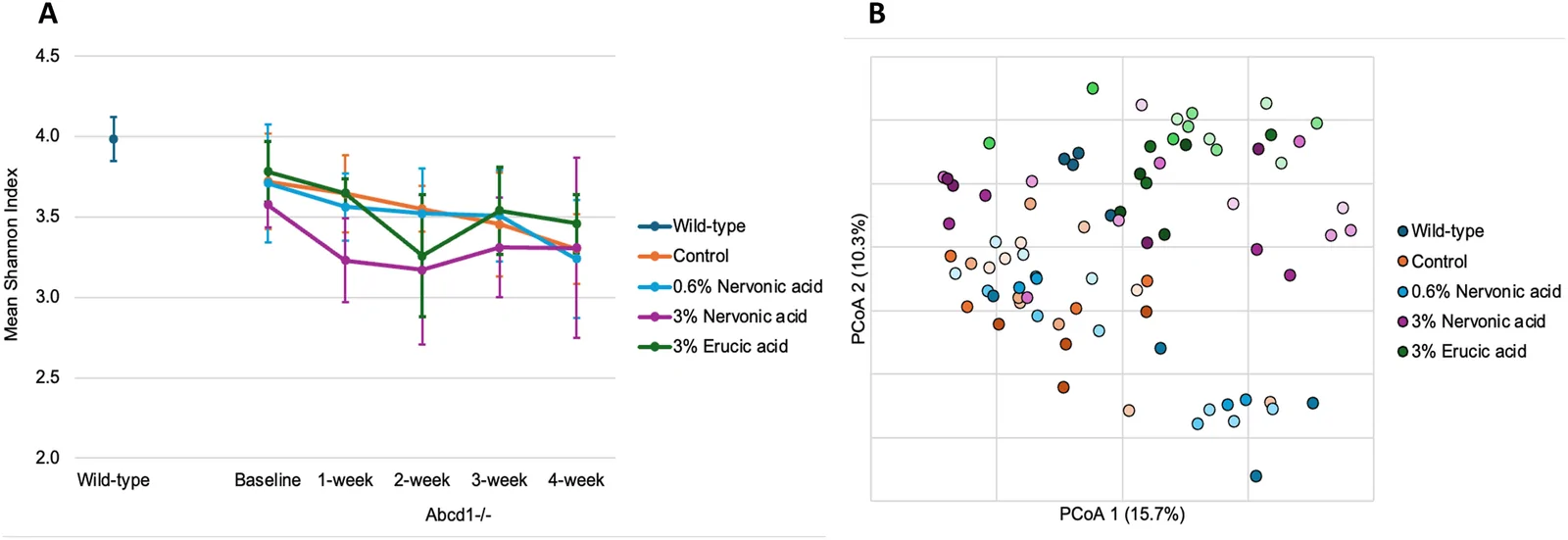
Diversity of microbial communities among all samples. **(A)** Alpha diversity as determined by the Shannon index, **(B)** Principal coordinate analysis (PCoA) of Bray-Curtis dissimilarities among all samples (R^2^ = 0.54). Error bars reflect standard deviation. Numbers within parentheses indicate the variation explained by the axis. Shading indicates the time point on the PCoA, with later samples shown in darker colors.

Longitudinal analysis of relative abundances of genera (or the most resolved taxonomic level available) revealed only three taxa that differed significantly between control mice and 3% EA-treated mice. Relative abundances of families *Muribaculaceae* and *Lachnospiraceae*, which could not be further classified to genus, were significantly greater among 3% EA-treated mice (p = 0.026 and 0.001), while relative abundances of *Bifidobacterium* were lower in 3% EA-treated mice relative to controls (p = 0.039). No other groups had taxa that differed significantly from controls longitudinally over the study period (p ≥ 0.090).

Similar to baseline and endpoint comparisons, no significant differences in community structure were observed over time within the *Abcd1−/−* control, 0.6% NA, or 3% NA groups (ANOSIM R = −0.042 - 0.187, p ≥ 0.068; Supplementary Fig. 1). While differences in community structure were significant among the 3% EA group (R = 0.358, p < 0.001), pairwise comparisons were not significant at Bonferroni-adjusted α = 0.005; p ≥ 0.031. Correspondingly, more ASVs were differentially abundant at different time points in the 3% EA group (Supplementary Table 3), primarily reflected in changes in abundance among the Bacteroidales order.

## Discussion

To date, ALD lacks effective pharmacological therapies for patients across different clinical presentations. The incorporation of ALD into newborn screening programs has enabled earlier detection and created opportunities for timely intervention [37]. However, a positive diagnosis does not predict disease trajectory. Asymptomatic males may eventually progress to either AMN or cALD over time [7]. More than 80% of females with *ABCD1* mutations develop an AMN-like phenotype later in life, with frequency increasing with age [38]. In affected males, the cerebral inflammatory phenotype predominantly occurs in boys before age 11 (mean onset ∼7 years), and without intervention, approximately 50% die within 5 years of symptom onset [39], highlighting the urgent need for early and effective treatments.

Dietary monounsaturated fatty acids reduce saturated VLCFA accumulation in ALD patient-derived fibroblasts, potentially through inhibition of their biosynthesis [40]. Lorenzo's Oil, a 4:1 mixture of oleic acid (C18:1) and erucic acid (C22:1, EA), demonstrated efficacy in lowering plasma C26:0 by up to 76% in asymptomatic boys with ALD [41,42]. However, limited pharmacological understanding, safety concerns regarding EA-induced cardiotoxicity in rats, and the absence of a clear pathway for formal regulatory approval limited its clinical advancement in the United States [43], [44], [45]. Notably, one mouse in the EA diet group from our study developed marked cardiomegaly. Although causality cannot be established, given the potential individual or environmental variability, this finding raises concerns regarding possible EA-associated cardiotoxicity. Hence, there is an absolute need for safe and effective alternative therapies for all patients identified with *ABCD1* mutations.

NA is a major structural component of sphingomyelin in the central nervous system [46]. It possesses essential biological functions, including roles in brain development, myelin integrity, and remyelination, and shares structural similarity with Lorenzo's Oil [47]. Decreased NA-containing sphingolipids have been observed in postmortem ALD and multiple sclerosis brains, underscoring their potential role in demyelinating pathology [48]. Moreover, in patient-derived ALD fibroblasts, we demonstrated that beyond its biochemical effect in reducing saturated VLCFAs, NA modulates cellular bioenergetics in a concentration-dependent manner [13,14]. Collectively, these observations provide a strong rationale for its further development as a disease-modifying therapy for ALD to prevent neurological dysfunction.

NA exhibited noteworthy biochemical efficacy in our 4-week feeding study. Plasma levels of C26:0-LPC, the diagnostic biomarker used in newborn screening for ALD, were significantly reduced as early as one week after initiation of NA treatment and sustained throughout the study. Importantly, a recent lipidomic study in patients with *ABCD1* defect reported that increased plasma levels of C26:0 and C26:0-LPC were positively correlated with the presence of adrenal insufficiency and cALD, and greater spinal cord disease [49], highlighting the clinical relevance of our findings. Recent studies of leriglitazone, a peroxisome proliferator-activated receptor gamma agonist, have demonstrated benefits in slowing lesion progression in patients with cALD [[50], [51], [52]]. However, no corresponding improvements in plasma biomarkers have been reported, and its clinical benefits across all disease phenotypes remain unclear. To our knowledge, our study represents the first report showing that a dietary intervention can robustly decrease C26:0-LPC levels by more than 50% in both plasma and brain in an ALD preclinical model. Given that VLCFA accumulation is a hallmark of all *ABCD1* mutation carriers, NA has the potential to benefit the full spectrum of patients, with promise as a preventative therapy for presymptomatic individuals identified by newborn screening and those at risk of developing cerebral disease.

Another notable observation in our study is that both free C24:1 and C24:1-LPC levels were significantly elevated across most peripheral tissues, but not in the brain, and this pattern corresponded to levels of free C26:1 and C26:1-LPC. Despite this, brain levels of C26:0, total saturated VLCFAs, and C26:0-LPC levels were significantly reduced. These findings indicate that high brain accumulation of NA may not be needed to elicit substantial biochemical effects. Fatty acids cross the blood-brain barrier either by passive diffusion or through specific transport proteins [53]. Among these, esterification to a glycerol backbone, such as in the form of LPC, is one of the most efficient mechanisms for brain delivery [53]. A plausible explanation is that NA crosses the blood-brain barrier via active transport and undergoes rapid metabolism within glial cells such as oligodendrocytes and astrocytes [54]. Alternatively, NA penetration into the brain may be limited, however, correction of hepatic metabolism alone may be sufficient to reduce brain C26:0-LPC levels. Further studies are needed to clarify the mechanisms driving these brain-specific lipid dynamics and to define the metabolic fate of NA within the central nervous system. An additional factor to consider is the technical challenge of quantifying NA in the brain due to the high endogenous levels of fatty acids, a limitation that can only be overcome by employing isotope-labeling techniques to distinguish exogenous from endogenous pools.

We did not observe a significant change in C26:0 or total saturated VLCFA in the adrenal gland following NA or EA treatment. Notably, the *Abcd1−/−* mice used in this study do not develop adrenal insufficiency at any age and adrenal steroidogenic function remains preserved despite biochemical VLCFA accumulation, suggesting that the adrenal gland is not functionally impaired in this model [55]. Consequently, the absence of modulation of adrenal saturated VLCFAs should be interpreted within the context of model-specific limitations rather than as evidence of reduced therapeutic activity. Moreover, preclinical studies have demonstrated that even after complete bilateral adrenalectomy with minimal replacement support, some rodents can gradually reestablish corticosterone levels, indicating the presence of compensatory mechanisms that may preserve glucocorticoid response [56]. Such physiological resilience may further limit the sensitivity of the adrenal gland as a pharmacodynamic endpoint in this model. Therefore, studies using alternative preclinical models that more faithfully recapitulate human adrenal insufficiency would be necessary to better evaluate the potential impact of NA on adrenal pathology.

To date, no studies have systematically investigated gut microbiome composition, diversity, or functional alterations in patients with ALD. Here, we report for the first time a comparative analysis of fecal microbial communities in wild-type and *Abcd1−/−* mice, revealing that *Abcd1−/−* mice harbored significantly lower relative abundances of *Lactobacillus* and greater abundances of *Faecalibaculum* compared with wild-type mice. These findings suggest that the ALD pathology can contribute to distinct gut microbiome alterations, offering a novel perspective for evaluating future therapeutic candidates. Moreover, this raises an important question of whether dietary supplementation with NA could reverse or mitigate the observed microbiome alterations. A few preclinical studies have reported that NA treatment modulates gut microbiota composition by increasing the abundance of beneficial bacteria such as *Lactobacillus* and reducing inflammation-associated taxa in an Alzheimer's disease rat model [57], as well as selectively restoring the reduced microbial diversity observed in cerebral ischemia-reperfusion injury rats [58]. However, these findings should be interpreted with caution, considering the experimental design, sample size, and statistical analysis approach, and more rigorous studies are needed to support causal inferences from rodent models to human diseases [59]. On the other hand, elevated fecal NA levels have been observed in patients with inflammatory bowel disease, where they positively correlate with clinically established inflammatory markers including C-reactive protein and fecal calprotectin [60]. The elevation of NA may represent a byproduct of a compensatory response to inflammation, while the distinct roles of endogenous NA and exogenously administered NA under such conditions remain to be elucidated. We did not detect remarkable differences between treatment groups or within groups over time. This lack of statistical significance may be attributed to the relatively short treatment duration and limited sample size, which likely reduced the power to detect subtle microbiome changes. Despite this, we observed significant changes in the relative abundance of three microbial taxa between the control group and the EA-treated group, suggesting that dietary fatty acids can influence gut microbial composition. These preliminary observations highlight the need for more comprehensive studies with extended treatment periods and larger cohorts to investigate the role of gut microbiota in ALD pathology and longer-term impact of NA intervention on microbiome composition and diversity, which may further contribute to the amelioration of disease symptoms.

The *Abcd1−/−* mice recapitulate biochemical hallmark features of ALD, including lipid cleft inclusions in adrenocortical cells and elevated tissue levels of saturated VLCFAs [19], but do not develop cerebral pathology, with neurological and behavioral impairments typically appearing only after 15 months [61,62]. Consequently, further evaluation of NA in preclinical models that exhibit cerebral demyelination and neuroinflammation, such as the recently developed cALD mouse model [63], is warranted to assess its efficacy in mitigating neuropathological aspects of ALD. Furthermore, incorporating both male and female mice in future studies will be necessary to evaluate potential sex-dependent effects and strengthen the translational applicability of our findings. Despite these limitations, our findings provide promising initial support for the continued development of NA as a therapeutic candidate for ALD.

Recent efforts have focused on developing inhibitors of elongation of very-long-chain fatty acids protein 1 (ELOVL1), the key enzyme responsible for synthesizing saturated and monounsaturated VLCFAs [64], to lower VLCFA levels in ALD. Treatment with highly selective inhibitors in mice effectively reduced VLCFAs in both plasma and brain; however, single-nucleus sequencing revealed unanticipated pathway alterations, including enhanced brain stress responses and downregulated sterol-related processes in the CNS [65]. Furthermore, toxicology studies in rats and monkeys identified significant safety concerns, underscoring the need for careful evaluation of this strategy [66]. Given that ELOVL1 plays essential physiological roles in maintaining the epidermal barrier, regulating sphingolipid levels, and modulating immune function, it is not surprising that complete inhibition of this enzyme leads to unintended adverse effects [67,68]. In contrast, NA may offer a safer means of modulating ELOVL1 activity by acting as a competitive and reversible substrate rather than a direct enzyme inhibitor, thereby reducing saturated VLCFA burden while minimizing broader perturbations of lipid metabolic pathways.

A previous study using enzyme kinetic analysis demonstrated that Lorenzo's oil inhibits ELOVL1 via a mixed inhibition mechanism [69]. Based on structural similarity and overlap within the elongation pathway, we speculate that NA may interact with ELOVL1 in a comparable manner. Such an interaction could shift the lipid profile away from the accumulation of toxic saturated VLCFAs toward the production of unsaturated species. Consistent with this hypothesis, NA treatment resulted in a significant reduction in plasma and brain C26:0 and total saturated VLCFAs, accompanied by increased levels of C26:1. Moreover, in line with the underlying metabolic impairment in ALD, NA treatment did not elevate C22:1 levels, suggesting that fatty acids are preferentially directed into elongation pathways rather than β-oxidation. Taken together, these findings are consistent with a model in which NA may influence C26:0 elongation dynamics through interaction with ELOVL1, potentially favoring the formation of monounsaturated C26:1 species and thereby reducing saturated VLCFA burden (Fig. 7). However, the precise mechanism underlying the interaction between NA and ELOVL1 remains to be determined through direct enzymatic characterization.

**Fig. 7:**
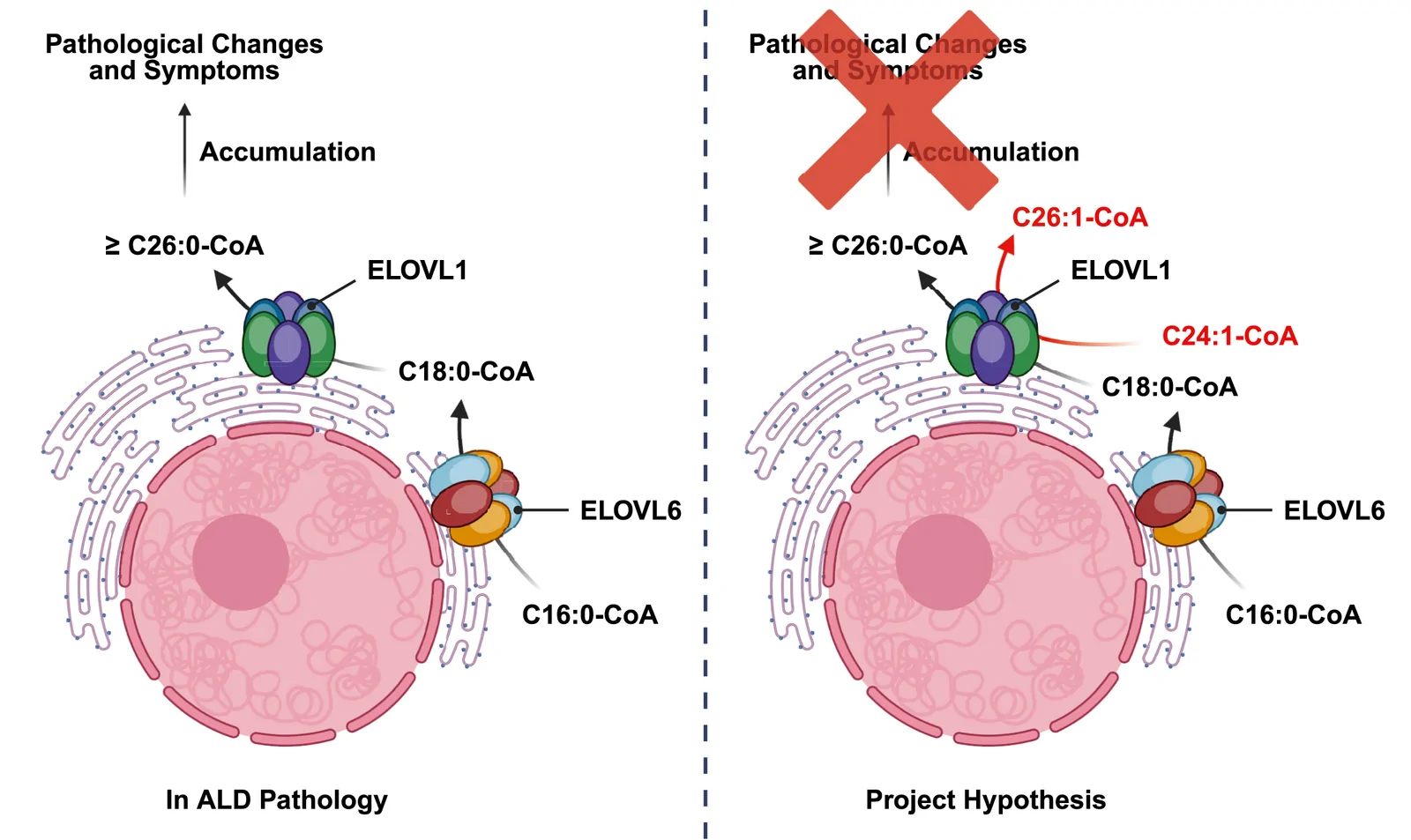
Schematic illustration of adrenoleukodystrophy (ALD) pathology and the proposed working hypothesis. C26:0 is synthesized via sequential elongation reactions catalyzed by elongase enzymes, of which elongation of very long-chain fatty acid protein 6 (ELOVL6) converts C16:0 to C18:0, followed by further elongation to C26:0 by ELOVL1. In ALD, mutations in the *ABCD1* gene result in a defective transporter responsible for importing VLCFAs into peroxisomes, leading to impaired peroxisomal β-oxidation and the subsequent accumulation of saturated VLCFAs (mainly C26:0) in plasma and tissues. We hypothesize that nervonic acid (NA, C24:1) can competitively bind to ELOVL1, thereby redirecting the elongation pathway toward the synthesis of monounsaturated C26:1 instead of saturated C26:0.

In conclusion, daily NA administration reduced saturated VLCFAs and their corresponding LPCs in both plasma and brain in a dose-dependent manner, without observable adverse effects, supporting a favorable safety profile in this preclinical model. These findings suggest that NA may represent a first-in-class, potentially preventative therapy for ALD that targets the underlying biochemical defect and may modify downstream pathological processes. Future studies evaluating long-term safety, efficacy, functional outcomes, and optimized dosing will be critical to advance NA toward clinical translation. Ultimately, NA represents a promising therapeutic candidate that warrants further investigation to define its clinical potential as a safe and accessible treatment strategy for ALD.

## Author contributions

**Designed experiments**: CL, MRT, EN, SS, TCL, RVK.

**Performed experiments**: CL, KB, ABM, WF, ML, SH, AT, WD.

**Performed computational analysis**: CL, CS.

**Authored manuscript**: CL, ABM, CS, RVK.

**Edited manuscript**: CL, ABM, WF, CS, SS, TCL, RVK.

## Data availability

The data that support the findings of this study are available from the corresponding author upon reasonable request.

## Funding

This work was supported by the National Center for Advancing Translational Sciences of the National Institutes of Health (Award Number 1R21TR003941-01).

## Declaration of competing interest

The authors declare that they have no known competing financial interests or personal relationships that could have appeared to influence the work reported in this paper.

## References

[1] Kemp S., Pujol A., Waterham H.R., van Geel B.M., Boehm C.D., Raymond G.V. (2001). ABCD1 mutations and the X-linked adrenoleukodystrophy mutation database: role in diagnosis and clinical correlations. Hum Mutat.

[2] Turk B.R., Theda C., Fatemi A., Moser A.B. (2020). X-linked adrenoleukodystrophy: pathology, pathophysiology, diagnostic testing, newborn screening and therapies. Int J Dev Neurosci Off J Int Soc Dev Neurosci.

[3] Yska H.A.F., Engelen M., Bugiani M. (2024). The pathology of X-linked adrenoleukodystrophy: tissue specific changes as a clue to pathophysiology. Orphanet J Rare Dis.

[4] Galea E., Launay N., Portero-Otin M., Ruiz M., Pamplona R., Aubourg P. (2012). Oxidative stress underlying axonal degeneration in adrenoleukodystrophy: a paradigm for multifactorial neurodegenerative diseases?. Biochim Biophys Acta.

[5] Hein S., Schönfeld P., Kahlert S., Reiser G. (2008). Toxic effects of X-linked adrenoleukodystrophy-associated, very long chain fatty acids on glial cells and neurons from rat hippocampus in culture. Hum Mol Genet.

[6] Singh I., Pujol A. (2010). Pathomechanisms underlying X-adrenoleukodystrophy: a three-hit hypothesis. Brain Pathol Zurich Switz.

[7] Engelen M., Kemp S., de Visser M., van Geel B.M., Wanders R.J.A., Aubourg P. (2012). X-linked adrenoleukodystrophy (X-ALD): clinical presentation and guidelines for diagnosis, follow-up and management. Orphanet J Rare Dis.

[8] Huffnagel I.C., Dijkgraaf M.G.W., Janssens G.E., van Weeghel M., van Geel B.M., Poll-The B.T. (2019). Disease progression in women with X-linked adrenoleukodystrophy is slow. Orphanet J Rare Dis.

[9] van Geel B.M., Bezman L., Loes D.J., Moser H.W., Raymond G.V. (2001). Evolution of phenotypes in adult male patients with X-linked adrenoleukodystrophy. Ann Neurol.

[10] Gupta A.O., Raymond G., Pierpont E.I., Kemp S., McIvor R.S., Rayannavar A. (2022). Treatment of cerebral adrenoleukodystrophy: allogeneic transplantation and lentiviral gene therapy. Expert Opin Biol Ther.

[11] Chiesa R., Boelens J.J., Duncan C.N., Kühl J.-S., Sevin C., Kapoor N. (2022). Variables affecting outcomes after allogeneic hematopoietic stem cell transplant for cerebral adrenoleukodystrophy. Blood Adv.

[12] Eichler F., Duncan C.N., Musolino P.L., Lund T.C., Gupta A.O., De Oliveira S. (2024). Lentiviral gene therapy for cerebral adrenoleukodystrophy. N Engl J Med.

[13] Terluk M.R., Tieu J., Sahasrabudhe S.A., Moser A., Watkins P.A., Raymond G.V. (2022). Nervonic acid attenuates accumulation of very long-chain fatty acids and is a potential therapy for adrenoleukodystrophy. Neurother J Am Soc Exp Neurother.

[14] Li C., Terluk M.R., Kartha R.V. (2025). Nervonic acid, a long chain monounsaturated fatty acid, improves mitochondrial function in adrenomyeloneuropathy fibroblasts. Br J Pharmacol.

[15] Wang X., Li Z., Li X., Liu X., YingMao, Cao F. (2023). Integrated metabolomics and transcriptomics reveal the neuroprotective effect of nervonic acid on LPS-induced AD model mice. Biochem Pharmacol.

[16] Aihaiti M., Shi H., Liu Y., Hou C., Song X., Li M. (2023). Nervonic acid reduces the cognitive and neurological disturbances induced by combined doses of D-galactose/AlCl3 in mice. Food Sci Nutr.

[17] Ma S., Lue Z., Xu G., Ma Y., Yuan W., Huang Z. (2024). Nervonic acid improves depression like behaviors and demyelination of medial prefrontal cortex in chronic restraint stress mice. Biochem Biophys Res Commun.

[18] Zhang X., Wu D., Yin Z. (2024). Nervonic acid alleviates MPTP-induced Parkinson's disease via MEK/ERK pathway. Cell Mol Biol Noisy--Gd Fr.

[19] Lu J.F., Lawler A.M., Watkins P.A., Powers J.M., Moser A.B., Moser H.W. (1997). A mouse model for X-linked adrenoleukodystrophy. Proc Natl Acad Sci U S A.

[20] Vaia Y., Bruschi F., Tagi V.M., Tosi M., Montanari C., Zuccotti G. (2024). Microbiota gut-brain axis: implications for pediatric-onset leukodystrophies. Front Nutr.

[21] Morita M., Honda A., Kobayashi A., Watanabe Y., Watanabe S., Kawaguchi K. (2017). Effect of Lorenzo's oil on hepatic gene expression and the serum fatty acid level in abcd1-Deficient mice. JIMD Rep.

[22] Folch J., Lees M., Sloane Stanley G.H. (1957). A simple method for the isolation and purification of total lipides from animal tissues. J Biol Chem.

[23] Lagerstedt S.A., Hinrichs D.R., Batt S.M., Magera M.J., Rinaldo P., McConnell J.P. (2001). Quantitative determination of plasma c8-c26 total fatty acids for the biochemical diagnosis of nutritional and metabolic disorders. Mol Genet Metab.

[24] Hubbard W.C., Moser A.B., Tortorelli S., Liu A., Jones D., Moser H. (2006). Combined liquid chromatography-tandem mass spectrometry as an analytical method for high throughput screening for X-linked adrenoleukodystrophy and other peroxisomal disorders: preliminary findings. Mol Genet Metab.

[25] Hubbard W.C., Moser A.B., Liu A.C., Jones R.O., Steinberg S.J., Lorey F. (2009). Newborn screening for X-linked adrenoleukodystrophy (X-ALD): validation of a combined liquid chromatography-tandem mass spectrometric (LC-MS/MS) method. Mol Genet Metab.

[26] van de Beek M.-C., Dijkstra I.M.E., van Lenthe H., Ofman R., Goldhaber-Pasillas D., Schauer N. (2016). C26:0-Carnitine is a new biomarker for X-Linked adrenoleukodystrophy in mice and man. PLoS One.

[27] Schloss P.D., Westcott S.L., Ryabin T., Hall J.R., Hartmann M., Hollister E.B. (2009). Introducing mothur: open-source, platform-independent, community-supported software for describing and comparing microbial communities. Appl Environ Microbiol.

[28] Staley C., Kaiser T., Vaughn B.P., Graiziger C.T., Hamilton M.J., Rehman T.U. (2018). Predicting recurrence of Clostridium difficile infection following encapsulated fecal microbiota transplantation. Microbiome.

[29] Pruesse E., Quast C., Knittel K., Fuchs B.M., Ludwig W., Peplies J. (2007). SILVA: a comprehensive online resource for quality checked and aligned ribosomal RNA sequence data compatible with ARB. Nucleic Acids Res.

[30] Edgar R.C., Haas B.J., Clemente J.C., Quince C., Knight R. (2011). UCHIME improves sensitivity and speed of chimera detection. Bioinforma Oxf Engl.

[31] Westcott S.L., Schloss P.D. (2017). OptiClust, an improved method for assigning amplicon-based sequence data to operational taxonomic units. mSphere.

[32] Cole J.R., Wang Q., Cardenas E., Fish J., Chai B., Farris R.J. (2009). The ribosomal database project: improved alignments and new tools for rRNA analysis. Nucleic Acids Res.

[33] Segata N., Izard J., Waldron L., Gevers D., Miropolsky L., Garrett W.S. (2011). Metagenomic biomarker discovery and explanation. Genome Biol.

[34] Clarke K.R. (1993). Non-parametric multivariate analyses of changes in community structure. Aust J Ecol.

[35] Shields-Cutler R.R., Al-Ghalith G.A., Yassour M., Knights D. (2018). SplinectomeR enables group comparisons in longitudinal microbiome studies. Front Microbiol.

[36] Body weight info - B6J (000664) | The Jackson Laboratory. https://www.jax.org/jax-mice-and-services/strain-data-sheet-pages/body-weight-chart-000664 (accessed 27 November 2025).

[37] Ramirez Alcantara J., Grant N.R., Sethuram S., Nagy A., Becker C., Sahai I. (2023). Early detection of adrenal insufficiency: the impact of newborn screening for adrenoleukodystrophy. J Clin Endocrinol Metab.

[38] Yan C., Pierpont E.I., Fine A.S., Kartha R.V. (2026). Clinically relevant outcome measures in women with adrenoleukodystrophy. Ann Clin Transl Neurol.

[39] Mahmood A., Raymond G.V., Dubey P., Peters C., Moser H.W. (2007). Survival analysis of haematopoietic cell transplantation for childhood cerebral X-linked adrenoleukodystrophy: a comparison study. Lancet Neurol.

[40] Rizzo W.B., Watkins P.A., Phillips M.W., Cranin D., Campbell B., Avigan J. (1986). Adrenoleukodystrophy: oleic acid lowers fibroblast saturated C22-26 fatty acids. Neurology.

[41] Rizzo W.B., Leshner R.T., Odone A., Dammann A.L., Craft D.A., Jensen M.E. (1989). Dietary erucic acid therapy for X-linked adrenoleukodystrophy. Neurology.

[42] Ahmed M.A., Kartha R.V., Brundage R.C., Cloyd J., Basu C., Carlin B.P. (2016). A model-based approach to assess the exposure-response relationship of Lorenzo's oil in adrenoleukodystrophy. Br J Clin Pharmacol.

[43] van Geel B.M., Assies J., Haverkort E.B., Koelman J.H., Verbeeten B., Wanders R.J. (1999). Progression of abnormalities in adrenomyeloneuropathy and neurologically asymptomatic X-linked adrenoleukodystrophy despite treatment with ‘Lorenzo's oil’. J Neurol Neurosurg Psychiatry.

[44] Aubourg P., Adamsbaum C., Lavallard-Rousseau M.C., Rocchiccioli F., Cartier N., Jambaqué I. (1993). A two-year trial of oleic and erucic acids (‘Lorenzo's oil’) as treatment for adrenomyeloneuropathy. N Engl J Med.

[45] Charlton K.M., Corner A.H., Davey K., Kramer J.K., Mahadevan S., Sauer F.D. (1975). Cardiac lesions in rats fed rapeseed oils. Can J Comp Med Rev Can Med Comp.

[46] Martínez M., Mougan I. (1998). Fatty acid composition of human brain phospholipids during normal development. J Neurochem.

[47] Li Q., Chen J., Yu X., Gao J.-M. (2019). A mini review of nervonic acid: source, production, and biological functions. Food Chem.

[48] Sargent J.R., Coupland K., Wilson R. (1994). Nervonic acid and demyelinating disease. Med Hypotheses.

[49] Jaspers Y.R.J., Yska H.A.F., Bergner C.G., Dijkstra I.M.E., Huffnagel I.C., Voermans M.M.C. (2024). Lipidomic biomarkers in plasma correlate with disease severity in adrenoleukodystrophy. Commun Med.

[50] García-Cazorla Á., Sevin C., Constante J.R., Yazbeck E., Rosewich H., Jimenez S. (2025). Safety and efficacy of leriglitazone in childhood cerebral adrenoleukodystrophy (NEXUS): an interim analysis of an open-label, phase 2/3 trial. eClinicalMedicine.

[51] Golse M., Weinhofer I., Blanco B., Barbier M., Yazbeck E., Huiban C. (2024). Leriglitazone halts disease progression in adult patients with early cerebral adrenoleukodystrophy. Brain.

[52] Köhler W., Engelen M., Eichler F., Lachmann R., Fatemi A., Sampson J. (2023). Safety and efficacy of leriglitazone for preventing disease progression in men with adrenomyeloneuropathy (ADVANCE): a randomised, double-blind, multi-centre, placebo-controlled phase 2–3 trial. Lancet Neurol.

[53] Pifferi F., Laurent B., Plourde M. (2021). Lipid transport and metabolism at the blood-brain interface: implications in health and disease. Front Physiol.

[54] Hofmann K., Rodriguez-Rodriguez R., Gaebler A., Casals N., Scheller A., Kuerschner L. (2017). Astrocytes and oligodendrocytes in grey and white matter regions of the brain metabolize fatty acids. Sci Rep.

[55] Lu J.-F., Barron-Casella E., Deering R., Heinzer A.K., Moser A.B., deMesy Bentley K.L. (2007). The role of peroxisomal ABC transporters in the mouse adrenal gland: the loss of Abcd2 (ALDR), not Abcd1 (ALD), causes oxidative damage. Lab Investig J Tech Methods Pathol.

[56] Gotlieb N., Albaz E., Shaashua L., Sorski L., Matzner P., Rosenne E. (2018). Regeneration of functional adrenal tissue following bilateral adrenalectomy. Endocrinology.

[57] Chen Z., Liu S., Song F., Hou Z., Zhou H., Fan Y. (2025). Integrated metabolome and microbiome strategy reveals the therapeutic effect of nervonic acid on Alzheimer's disease rats. J Nutr Biochem.

[58] Zhou H., Chen Z., Li J., Wang R., Bu H., Ruan C. (2024). Dietary supplementation with nervonic acid ameliorates cerebral ischemia-reperfusion injury by modulating of gut microbiota composition-fecal metabolites interaction. Mol Nutr Food Res.

[59] Walter J., Armet A.M., Finlay B.B., Shanahan F. (2020). Establishing or exaggerating causality for the gut microbiome: lessons from human microbiota-associated rodents. Cell.

[60] Kunst C., Elger T., Loibl J., Huss M., Kandulski A., Krautbauer S. (2024). Fecal nervonic acid as a biomarker for diagnosing and monitoring inflammatory bowel disease. Biomedicines.

[61] Pujol A., Hindelang C., Callizot N., Bartsch U., Schachner M., Mandel J.L. (2002). Late onset neurological phenotype of the X-ALD gene inactivation in mice: a mouse model for adrenomyeloneuropathy. Hum Mol Genet.

[62] Nemeth C.L., Gӧk Ö., Tomlinson S.N., Sharma A., Moser A.B., Kannan S. (2023). Targeted brain delivery of Dendrimer-4-Phenylbutyrate ameliorates neurological deficits in a long-term ABCD1-Deficient mouse model of X-Linked adrenoleukodystrophy. Neurother J Am Soc Exp Neurother.

[63] Hashemi E., Srivastava I.N., Aguirre A., Yoseph E.T., Kaushal E., Awani A. (2025). A novel mouse model for cerebral inflammatory demyelination in X-linked adrenoleukodystrophy: insights into pathogenesis and potential therapeutic targets. Ann Neurol.

[64] Kihara A. (2012). Very long-chain fatty acids: elongation, physiology and related disorders. J Biochem (Tokyo).

[65] Huang J.Y., Freed B., Hanus M., Keefe K., Chiang M.S.R., Brezzani A. (2025). Elovl1 inhibition reduced very long chain fatty acids in a mouse model of adrenoleukodystrophy. iScience.

[66] Boyd M.J., Collier P.N., Clark M.P., Deng H., Kesavan S., Ronkin S.M. (2021). Discovery of novel, orally bioavailable pyrimidine ether-based inhibitors of ELOVL1. J Med Chem.

[67] Wang X., Yu H., Gao R., Liu M., Xie W. (2023). A comprehensive review of the family of very-long-chain fatty acid elongases: structure, function, and implications in physiology and pathology. Eur J Med Res.

[68] Siddiqui A.J., Jahan S., Chaturvedi S., Siddiqui M.A., Alshahrani M.M., Abdelgadir A. (2023). Therapeutic role of ELOVL in neurological diseases. ACS Omega.

[69] Sassa T., Wakashima T., Ohno Y., Kihara A. (2014). Lorenzo's oil inhibits ELOVL1 and lowers the level of sphingomyelin with a saturated very long-chain fatty acid. J Lipid Res.

