# Supplementary material for: Nervonic acid supplementation mitigates disease severity biomarkers in adrenoleukodystrophy
Source: Neurotherapeutics. 2026 Apr 7;23(4):e00890. doi: 10.1016/j.neurot.2026.e00890 (PMC13639632; doi:10.1016/j.neurot.2026.e00890)
Supplement: Supplementary file 1 [file mmc1.docx]

**Supplementary Figure 1.** *Distribution of predominant genera among treatment groups at each time point.* ***(A)*** *Abcd1-/- control mice,* ***(B)*** *mice treated with 0.6% Nervonic acid,* ***(C)*** *mice treated with 3% Nervonic acid, and* ***(D)*** *mice treated with 3% Erucic acid. Error bars reflect standard deviation. Less abundant genera were consolidated for clarity. (f) indicates taxon could not be further classified beyond family.*

**Supplementary Table 1.** Linear discriminant analysis (LDA) of effect size (LEfSE) analysis of ASVs that varied significantly among treatments at baseline (LDA ≥ 3.0).

| **Treatment** | **Taxon** | **LDA** | ***P*-value** |
| --- | --- | --- | --- |
| **Wild-type** | *Lactobacillus* | 4.97 | 0.02 |
|  | *Bacteroidales* unclassified | 4.30 | 0.01 |
|  | *Lachnospiraceae* unclassified | 4.17 | 0.02 |
|  | *Bacteroidetes* unclassified | 4.13 | 0.00 |
|  | *Bacteroides* | 4.04 | 0.02 |
|  | *Alistipes* | 3.91 | 0.02 |
|  | *Phocaeicola* | 3.90 | 0.00 |
|  | *Lachnospiraceae* unclassified | 3.87 | 0.00 |
|  | *Muribaculaceae* unclassified | 3.86 | 0.00 |
|  | *Lachnospiraceae* unclassified | 3.81 | 0.04 |
|  | *Lachnospiraceae* unclassified | 3.77 | 0.04 |
|  | *Muribaculaceae* unclassified | 3.69 | 0.03 |
|  | *Bacteroidales* unclassified | 3.58 | 0.00 |
|  | *Ruminococcaceae* unclassified | 3.53 | 0.00 |
|  | *Alistipes* | 3.50 | 0.00 |
|  | *Ruminococcaceae* unclassified | 3.44 | 0.03 |
|  | *Ruminococcaceae* unclassified | 3.29 | 0.04 |
|  | *Paramuribaculum* | 3.29 | 0.02 |
|  | *Duncaniella* | 3.23 | 0.01 |
|  | *Staphylococcus* | 3.08 | 0.01 |
|  | *Alistipes* | 3.03 | 0.02 |
|  | *Eggerthellaceae* unclassified | 3.01 | 0.03 |
|  | *Lachnospiraceae* unclassified | 3.01 | 0.00 |
| **Control** | *Muribaculaceae* unclassified | 4.23 | 0.00 |
|  | *Romboutsia* | 4.03 | 0.03 |
|  | *Turicibacter* | 4.00 | 0.00 |
|  | *Muribaculum* | 3.89 | 0.01 |
|  | *Duncaniella* | 3.88 | 0.03 |
|  | *Muribaculaceae* unclassified | 3.83 | 0.00 |
|  | *Lachnospiraceae* unclassified | 3.72 | 0.03 |
|  | *Anaeroplasma* | 3.69 | 0.02 |
|  | *Bacteria* unclassified | 3.56 | 0.01 |
|  | *Lachnospiraceae* unclassified | 3.42 | 0.03 |
|  | *Sutterellaceae* unclassified | 3.32 | 0.02 |
|  | *Bacteria* unclassified | 3.26 | 0.01 |
| **0.6% Nervonic acid** | *Faecalibaculum* | 5.01 | 0.04 |
|  | *Bifidobacterium* | 4.65 | 0.03 |
|  | *Lachnospiraceae* unclassified | 4.15 | 0.00 |
|  | *Helicobacter* | 3.96 | 0.00 |
|  | *Parabacteroides* | 3.64 | 0.00 |
|  | *Lachnospiraceae* unclassified | 3.57 | 0.00 |
|  | *Lachnospiraceae* unclassified | 3.43 | 0.04 |
|  | *Odoribacter* | 3.42 | 0.02 |
|  | *Lachnospiraceae* unclassified | 3.41 | 0.00 |
|  | *Lachnospiraceae* unclassified | 3.34 | 0.00 |
| **3% Nervonic acid** | *Bacteroides* | 4.58 | 0.04 |
|  | *Lachnospiraceae* unclassified | 4.30 | 0.00 |
|  | *Duncaniella* | 4.04 | 0.00 |
|  | *Parabacteroides* | 3.90 | 0.01 |
|  | *Prevotellaceae* unclassified | 3.73 | 0.02 |
|  | *Helicobacter* | 3.72 | 0.01 |
|  | *Muribaculaceae* unclassified | 3.71 | 0.00 |
|  | *Phocaeicola* | 3.67 | 0.04 |
|  | *Paramuribaculum* | 3.56 | 0.01 |
|  | *Odoribacter* | 3.52 | 0.01 |
|  | *Lachnospiraceae* unclassified | 3.49 | 0.03 |
|  | *Alistipes* | 3.39 | 0.04 |
|  | *Adlercreutzia* | 3.27 | 0.02 |
|  | *Lachnospiraceae* unclassified | 3.21 | 0.03 |
| **3% Erucic acid** | *Muribaculaceae* unclassified | 4.56 | 0.01 |
|  | *Paramuribaculum* | 4.34 | 0.03 |
|  | *Duncaniella* | 4.02 | 0.00 |
|  | *Dorea* | 3.99 | 0.01 |
|  | *Lachnospiraceae* unclassified | 3.90 | 0.01 |
|  | *Muribaculaceae* unclassified | 3.88 | 0.03 |
|  | *Lachnospiraceae* unclassified | 3.70 | 0.01 |
|  | *Clostridium_XlVa* | 3.69 | 0.01 |
|  | *Lachnospiraceae* unclassified | 3.56 | 0.01 |
|  | *Duncaniella* | 3.44 | 0.01 |
|  | *Coriobacteriia* unclassified | 3.38 | 0.01 |
|  | *Coriobacteriia* unclassified | 3.35 | 0.00 |
|  | *Coriobacteriia* unclassified | 3.16 | 0.01 |
|  | *Olsenella* | 3.14 | 0.03 |

**Supplementary Table 2.** LEfSE analysis of ASVs that varied significantly among treatments at endpoint (LDA ≥ 3.0).

| **Treatment** | **Taxon** | **LDA** | ***P*-value** |
| --- | --- | --- | --- |
| **Control** | *Turicibacter* | 4.89 | 0.04 |
|  | *Bacteria* unclassified | 3.76 | 0.00 |
|  | *Clostridiales* unclassified | 3.62 | 0.03 |
| **0.6% Nervonic acid** | *Ligilactobacillus* | 3.12 | 0.02 |
|  | *Lachnospiraceae* unclassified | 4.40 | 0.02 |
|  | *Helicobacter* | 3.61 | 0.01 |
|  | *Acetatifactor* | 3.27 | 0.01 |
| **3% Erucic acid** | *Muribaculaceae* unclassified | 3.33 | 0.01 |
|  | *Lachnospiraceae* unclassified | 4.48 | 0.04 |
|  | *Bacteroides* | 4.09 | 0.00 |
|  | *Bacteroides* | 4.08 | 0.05 |
|  | *Dorea* | 3.91 | 0.04 |
|  | *Muribaculaceae* unclassified | 3.40 | 0.01 |
|  | *Sutterellaceae* unclassified | 3.28 | 0.01 |
|  | *Lachnospiraceae* unclassified | 3.06 | 0.03 |
|  | *Clostridium_XlVa* | 3.57 | 0.04 |

**Supplementary Table 3.** LEfSE analysis of ASVs that varied significantly over time within a treatment group (LDA ≥ 3.0). Taxonomic annotations are shown to genus level where possible.

| **Treatment** | **Time point** | **Taxon** | **LDA** | ***P*-value** |
| --- | --- | --- | --- | --- |
| **Control** | Baseline | *Dorea* | 3.38 | 0.01 |
|  | Baseline | *Lachnospiraceae* unclassified | 3.08 | 0.03 |
|  | Baseline | *Lachnospiraceae* unclassified | 3.01 | 0.03 |
|  | 1-week | *Ligilactobacillus* | 4.83 | 0.02 |
| **0.6% Nervonic acid** | Baseline | *Muribaculaceae* unclassified | 4.30 | 0.05 |
|  | Baseline | *Romboutsia* | 4.03 | 0.02 |
|  | Baseline | *Parabacteroides* | 3.36 | 0.01 |
|  | Baseline | *Alphaproteobacteria* unclassified | 3.01 | 0.01 |
|  | 1-week | *Duncaniella* | 3.32 | 0.04 |
|  | 2-week | *Muribaculaceae* unclassified | 3.55 | 0.02 |
|  | 4-week | *Ligilactobacillus* | 5.23 | 0.03 |
| **3% Nervonic acid** | Baseline | *Parabacteroides* | 3.93 | 0.05 |
|  | Baseline | *Duncaniella* | 3.71 | 0.02 |
|  | Baseline | *Alistipes* | 3.51 | 0.04 |
|  | 1-week | *Duncaniella* | 3.72 | 0.04 |
|  | 1-week | *Parabacteroides* | 3.15 | 0.05 |
| **3% Erucic acid** | Baseline | *Muribaculaceae* unclassified | 4.51 | 0.04 |
|  | Baseline | *Paramuribaculum* | 4.31 | 0.02 |
|  | Baseline | *Bacteroidales* unclassified | 3.92 | 0.05 |
|  | Baseline | *Paramuribaculum* | 3.84 | 0.04 |
|  | Baseline | *Duncaniella* | 3.83 | 0.04 |
|  | Baseline | *Dorea* | 3.64 | 0.03 |
|  | Baseline | *Clostridium XlVa* | 3.53 | 0.01 |
|  | Baseline | *Parabacteroides* | 3.51 | 0.01 |
|  | Baseline | *Coriobacteriia* unclassified | 3.32 | 0.05 |
|  | Baseline | *Lachnospiraceae* unclassified | 3.31 | 0.00 |
|  | Baseline | *Alistipes* | 3.14 | 0.01 |
|  | 1-week | *Ruminococcaceae* unclassified | 3.59 | 0.03 |
|  | 3-week | *Bacteroides* | 4.03 | 0.05 |
|  | 3-week | *Phocaeicola* | 3.65 | 0.03 |
|  | 3-week | *Parabacteroides* | 3.53 | 0.05 |
|  | 3-week | *Muribaculaceae* unclassified | 3.41 | 0.03 |
|  | 4-week | *Faecalibaculum* | 4.92 | 0.04 |
